# From the ESGE Editor

**Published:** 2019-03

**Authors:** Ertan Saridogan

A new era is starting in the history of both the European Society for Gynaecological Endoscopy (ESGE) and Facts, Views and Vision. This partnership will create a publishing platform to provide scientific facts, to debate on controversial or well established practices, and to propose future treatments and research. It will bring together the experience built by the journal and the society over the years to achieve this task.

The official journal of the ESGE, Facts, Views and Vision (FV&V) opens its doors to the clinicians, academics and researchers to publish original articles, guidelines, commentaries and opinion articles. It is impossible to think of an endoscopic journal without the use of video technology, hence video articles will be a regular feature in the new journal.

In this issue of FV&V, we have a combination of these features. The British Society for Gynaecological Endoscopy (BSGE) Guideline on Laparoscopy in Pregnancy by Ball et al. cover a very relevant topic for the gynaecological endoscopist surgeons. Abdominal and pelvic surgery during pregnancy is a challenging task and decisions are usually made after hard thinking. The guideline was commissioned by the BSGE and devised using the methodology of Royal College of Obstetricians and Gynaecologists for evidence based guidelines. They concluded that only surgeons who have adequate laparoscopic skills and who perform complex laparoscopic surgery regularly should undertake laparoscopy in pregnant women. There is no doubt that the guideline will be an important resource for clinicians in years to come.

Also, the FV&V journal will facilitate structured training programmes for surgical procedures. Such an example is in the article by Rusch et al. which proposes a standardised educational programme for robot assisted gynaecological surgery. The authors, are all experts in this field, nominated by the Society of European Robotic Gynaecological Surgery followed a Delphi process to develop recommendations on a stepwise training and assessment programme. This consensus document is likely to be a main reference point for training centres and trainees seeking to achieve competence in robotic gynaecological surgery.

Endometriosis is frequently described as an enigmatic condition. Philippe Koninckx and his colleagues, who have many years of experience between them in this field, eloquently describe the variability amongst women with this condition and highlight the limitations of traditional statistical analysis in detecting the hidden subgroups. They propose paying special attention to the extremes of an analysis and individual data.

As such, the new FV&V is an ambitious open access journal which promises to deliver multifaceted benefits. Its first issue, hereby, is an initial step to fulfilling this task. Whilst this issue includes input from the European societies, we expect to provide material from further afield, through a worldwide network of gynaecological endoscopists.

I hope you enjoy reading and utilising the material in this issue and look forward to delivering the next one.

Ertan Saridogan ESGE Editor

